# The developing immune network in human prenatal skin

**DOI:** 10.1111/imm.13192

**Published:** 2020-04-14

**Authors:** Rachel Anne Botting, Muzlifah Haniffa

**Affiliations:** ^1^ Faculty of Medical Sciences Biosciences Institute Newcastle University Newcastle upon Tyne UK; ^2^ Wellcome Sanger Institute Hinxton UK; ^3^ Department of Dermatology and NIHR Newcastle Biomedical Research Centre Newcastle Hospitals NHS Foundation Trust Newcastle upon Tyne UK

**Keywords:** haematopoiesis, immune homeostasis, regulation/suppression, skin

## Abstract

Establishment of a well‐functioning immune network in skin is crucial for its barrier function. This begins *in utero* alongside the structural differentiation and maturation of skin, and continues to expand and diversify across the human lifespan. The microenvironment of the developing human skin supports immune cell differentiation and has an overall anti‐inflammatory profile. Immunologically inert and skewed immune populations found in developing human skin promote wound healing, and as such may play a crucial role in the structural changes occurring during skin development.

## Graphical abstract

The immune system in skin is established early during development. Throughout development it continues to expand and diversify.

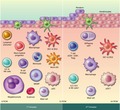

**Abbreviations**
APC: antigen‐presenting cell
BMP7: bone morphogenic protein 7
CLA: cutaneous lymphocyte antigen
DC: dendritic cell
ILC: innate lymphoid cell
KLR: killer cell lectin receptor
LC: Langerhans cell
MEMP: megakaryocyte‐erythrocyte‐mast cell progenitor
NK: natural killer cell
PCW: post‐conception week
RANK(L): receptor activator or nuclear factor‐kappaB (ligand)
RUNX: Runt‐related transcription factor 3
scRNAseq: single‐cell RNA sequencing
TGF‐β1: transforming growth factor beta 1
Treg: regulatory T‐cell

## The developing human skin

**Figure 1:**
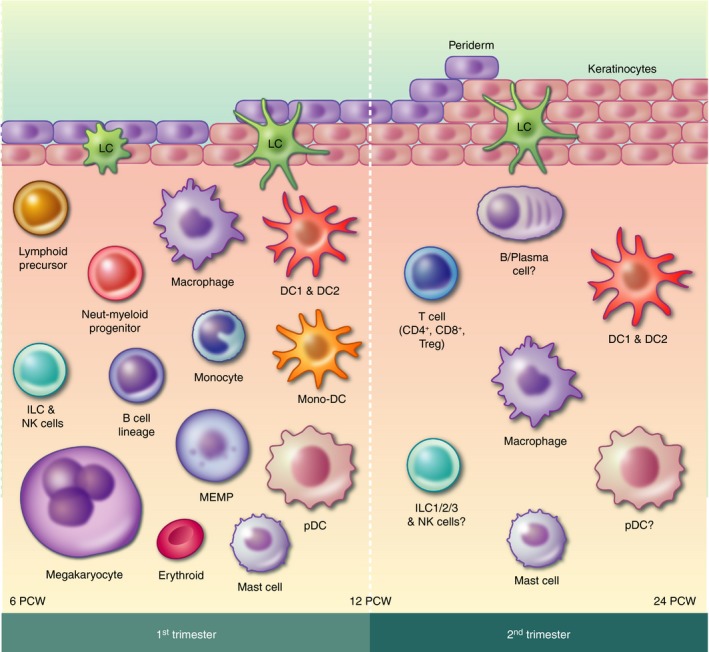
Overview of the immune cells present in first‐ and second‐trimester human skin. ?, unknown; DC, dendritic cell; ILC, innate lymphoid cell; LC, Langerhans cell; MEMP, megakaryocyte‐erythroid‐mast cell progenitor; Mono‐DC, monocyte‐DC hybrid; Neut‐myeloid progenitor, neutrophil‐myeloid progenitor; NK, natural killer cell.

The human skin undergoes significant structural changes during development *in utero* into a structurally complex organ in postnatal life and adulthood. The two major layers of human skin, the epidermis and dermis, develop over different gestational stages.^1^, ^2^, ^3^ The epidermis starts as a monolayer, which gains a superficial layer called the periderm within 4 weeks.^2^ The epidermis begins to stratify at the end of the first trimester, and consists of definitive layers towards the end of the second trimester.^1^, ^2^, ^3^, ^4^, ^5^ The dermis is initially densely cellular, which decreases after 8 post‐conception weeks (PCW) following increased production of extracellular matrix components.^1^, ^4^ The epidermis and dermis interact throughout the development process, and this is most evident during skin appendage formation, which begins at the start of the second trimester.^3^, ^4^, ^5^ While structural maturation is complete by birth, skin continues to functionally mature into adulthood.^6^ In tandem to the major structural changes occurring during the development of skin, the immune composition expands in number and diversity. Despite containing significantly fewer immune cells compared with mature adult skin,^7^, ^8^, ^9^, ^10^ the developing skin supports a diverse range of immune populations (Fig. 1).

As haematopoiesis occurs sequentially in the yolk sac, fetal liver and bone marrow, immune cells seeding skin could arise from any of these three sites of developmental haematopoiesis. The postnatal persistence and functional relevance of immune cells from yolk sac and fetal liver in humans remains undefined. In addition, the precise role of immune cells in what is currently presumed a sterile environment *in utero* is unclear, but there is increasing support for the role of immune cells in tissue generation and regeneration. Our review highlights the immune cells in human skin and their role in the development of skin as a barrier organ.

## Antigen‐presenting cells

### Langerhans cells

Specific to stratified squamous epithelium, Langerhans cells (LCs) are of particular interest when studying developing skin and its immune environment. The epidermis undergoes significant structural changes throughout development, during which LCs take up residence as early as 6 PCW.^11^ In mice, skin LCs are of dual origin, arising from yolk sac macrophages and fetal liver monocytes,^12^, ^13^, ^14^, ^15^, ^16^ and whether the same is true for LCs in human skin remains unknown. LCs can also be found in type II mucosal sites, such as the oral mucosa and vagina; however, in mice these originate from bone marrow precursors.^17^, ^18^ In skin, LCs are seeded during embryogenesis, and in mice have been shown to contribute to the population of LCs residing in adult skin,^12^ while mucosal LCs are seeded after birth and are continuously replenished from circulating bone marrow‐derived precursors.^12^, ^17^, ^18^, ^19^, ^20^ However, during inflammation it has been shown in mice that monocytes can differentiate into skin LCs,^21^, ^22^, ^23^ and in humans after severe depletion skin LCs can be reconstituted from donor haematopoietic stem cell‐derived precursors following bone marrow transplantation.^24^ Redundancy in LC establishment in skin through multiple origins, along with alternate pathways for reconstitution after depletion, is likely a reflection of their importance in skin. Furthermore, establishment during embryogenesis, rather than postnatal seeding, suggests a functional role for LCs in the developing skin.

LCs go through morphological and phenotypic changes within skin; they grow larger, more dendritic, gain expression of Langerin and CD1a, and form Birbeck granules in a stepwise manner.^7^, ^11^, ^25^ LCs are uniformly dendritic from 8 PCW,^25^ while surface expression and Birbeck granule formation continue to develop into the second trimester.^7^, ^11^, ^25^ Langerin expression is not detected prior to 9 PCW, and precedes CD1a expression, which is consistently seen from 11 PCW,^7^, ^11^ although rare CD1a^+^ epidermal cells have been detected from as early as 8 PCW.^25^ Similarly, expression of receptor activator of nuclear factor‐kappaB (RANK) by LCs is acquired gradually, with weak expression detected towards the end of the first trimester, and adult levels reached by mid‐gestation.^26^ Birbeck granules develop during the second trimester, showing heterogeneity within the LC population until about 16 PCW, from whence they resemble adult LCs.^25^ Heterogeneity is also observed in expression of HLA‐DR, CD36 and CD1c,^25^, ^27^ which may be due to differences in growth factors as the cells differentiate.

Differentiation of LCs occurs within skin and is supported by its microenvironment (for review, see Strobl *et al*.^28^). The absence of langerin expression in fetal dermis^7^, ^8^ suggests that LC differentiation is restricted to the epidermis as opposed to migration of mature LCs into the epidermis occurring. Transforming growth factor beta 1 (TGF‐β1), bone morphogenic protein 7 (BMP7), RANK ligand (RANKL) and IL‐34 produced by keratinocytes have been shown in mice to be important during LC growth and differentiation.^29^, ^30^, ^31^, ^32^, ^33^, ^34^ TGF‐β1 and BMP7 can be used to culture LC‐like cells from human monocytes and CD34 progenitors *in vitro*,^31^, ^35^ and RANKL supported their survival.^34^ TGF‐β1 has also been linked to Runt‐related transcription factor 3 (RUNX3) expression, which has been shown in mouse and *in vitro* human culture systems to be an important positive regulator of LC differentiation.^36^, ^37^ TGF‐β1 precursor protein and BMP7 can be detected from 6 PCW in human embryonic epidermis,^7^, ^31^ and the active form of TGF‐β1 can be detected from 7 PCW.^7^ Despite showing weaker staining, the pattern of TGF‐β1 expression in fetal skin resembles that seen in adults by the end of the second trimester,^7^ which coincides with LCs in fetal skin being phenotypically indistinguishable from those in adult skin.^7^, ^25^ Although phenotypic maturity of LCs is reached at this stage, it remains uncertain how functionally mature the human fetal LCs are, or which intrinsic and extrinsic factors drive the maturation process *in vivo*.

### Dermal dendritic cells and macrophages

Unlike the epidermis, during development the dermis hosts multiple antigen‐presenting cell (APC) subsets. Mature adult skin contains the dendritic cell (DC) subsets DC1 and DC2, as well as macrophages, all of which have been identified in first‐trimester human skin and closely resemble their adult counterparts.^38^ Macrophages greatly outnumber DCs in the first trimester, but reach adult ratios mid‐gestation,^7^, ^39^ coinciding with bone marrow development.^40^ Furthermore, plasmacytoid DCs, monocytes, monocyte‐DCs (having both a monocyte and DC gene signature) and a neutrophil‐myeloid precursor have also recently been identified in first‐trimester human skin using single‐cell RNA sequencing (scRNAseq).^41^

Macrophages are the most abundant immune subset in fetal skin,^41^ which may elicit a multitude of functions due to their plasticity.^42^ As has been shown in mice, they may be derived from progenitors originating in yolk sac, fetal liver or bone marrow.^13^, ^16^, ^43^ Embryonic and fetal macrophages in the first‐ and second‐trimester express MR (CD206) and DC‐SIGN (CD209),^8^, ^39^ which is typically attributed to an M2 phenotype.^44^ M2 macrophages are anti‐inflammatory and support migration and differentiation of fibroblasts and keratinocytes during wound healing,^45^, ^46^ and in mice and zebrafish have been shown to support vascularization.^47^ As such, macrophages in developing human skin may play a crucial role in supporting the structural changes occurring during maturation of skin.

Unlike macrophages and LCs, very little is known about dermal DCs in developing skin beyond their presence. DC1 and DC2 have been identified in fetal skin prior to bone marrow production,^38^, ^39^, ^41^ as such they may originate from both fetal liver‐ and bone marrow‐derived precursors. Major Histocompatibility Complex class II^+^ cells have been observed in lymphatic vessels of second‐trimester skin,^38^ suggesting DCs may migrate as part of homeostatic surveillance. Splenic DCs from matched samples have been shown to induce T‐cell proliferation, but similar functional studies of fetal skin DCs have yet to be reported. Whether fetal liver‐ and bone marrow‐derived DCs found in fetal skin behave in a similar fashion, and whether they functionally resemble dermal DCs in healthy adult skin will be an interesting avenue to explore.

## Lymphoid cells

### T‐cells

Healthy adult skin hosts multiple T‐cell subsets and at proportions greater than those seen in circulation.^48^ The establishment of this skin T‐cell network begins in the second trimester,^9^, ^10^, ^39^ which coincides with the production and egress of mature T‐cells from the thymus into the circulation.^49^, ^50^, ^51^ Unlike adult skin, only αβ T‐cells have been observed in fetal skin,^39^, ^52^ despite γδ T‐cells being present in the circulation.^53^ Second trimester fetal skin contains 4–7% the abundance of T‐cells found in adult skin,^10^, ^39^, ^48^ and with γδ T‐cells representing 1–10% of the total T‐cells found in adult skin,^48^ the frequency may be too low for confident detection by flow cytometry or immunofluorescence. Other high‐resolution techniques, such as scRNAseq, may reveal the presence of these cells.

Fetal skin contains both CD4^+^ and CD8^+^ T‐cells, of which naïve CD4^+^ T‐cells are the most abundant and increase in frequency across development.^10^ In contrast to T‐cells in adult skin, which are predominantly memory‐effector/cutaneous lymphocyte antigen (CLA)^+^ T‐cells,^48^ fetal skin contains very few memory‐effector/CLA^+^ T‐cells, even into the third trimester.^10^ This may in part be due to lower levels of T‐cell skin‐homing cytokine CCL27^54^ in fetal skin compared with adult.^8^, ^39^, ^55^ CCL27 is predominantly expressed by differentiated keratinocytes toward the end of the second trimester, with little to no staining in the first trimester.^55^ As such, it has been suggested that seeding of naïve T‐cells in fetal skin occurs independently of CCL27.^39^ The structural and functional immaturity of the epidermis at the ages investigated may also explain why epidermal T‐cells have not yet been identified in fetal skin.^8^, ^10^, ^39^

Regulatory T‐cells (Tregs) constitute 10−20% of the total T‐cell pool in fetal skin, which is comparable to proportions observed in healthy adult skin.^9^, ^39^ Tregs in adult skin are preferentially localized to hair follicles,^9^ which in fetal skin do not develop until mid‐gestation^3^ and may explain the irregular localization of T‐cells in fetal skin.^10^ T‐cells from fetal mesenteric lymph nodes have been reported to be highly responsive to stimuli and, without active suppression from fetal Tregs, proliferate extensively.^56^ It would be interesting to determine whether T‐cells in fetal skin behave in a similar manner, what role they play, and whether the Tregs play an active role in suppressing these and other immune cells in skin.

### Innate lymphoid cells

While T‐cells were only detected in fetal skin after thymic production, natural killer (NK) cells and innate lymphoid cell (ILC) precursors have been identified in skin as early as 8 PCW.^41^ Despite being the most abundant immune cell types isolated from fetal skin after macrophages,^41^ these are otherwise poorly described. These NK cells expressed genes for perforin, killer cell lectin receptors (KLRs), interferon‐γ and multiple granzymes,^41^ showing resemblance to functionally mature NK cells. Ivarsson *et al*. phenotypically and functionally compared NK cells across fetal lung, spleen, liver, bone marrow and mesenteric lymph nodes, finding the NK cells in lung were more differentiated than the other sites.^57^ Similar to fetal skin, lungs are exposed to amniotic fluid, and after birth will be exposed to an array of potentially immunogenic factors from the external environment, therefore the NK cells in fetal skin may share a similar profile to those in fetal lung. In contrast to first‐trimester skin NK cells, the gene signature of the other ILCs is more similar to that of an undifferentiated ILC.^41^ Cells with a gene signature consistent with ILC2s were detected in second‐trimester skin by scRNAseq (Botting *et al*., unpublished), which may have differentiated from the ILC precursor found in first‐trimester skin. Therefore, this ILC precursor may also give rise to the ILC1, 2 and 3 subsets that have been described in healthy adult skin.^58^

As well as ILC precursors, lymphocyte precursors were also identified in first‐trimester skin by scRNAseq, where they were found to be more abundant in embryonic than fetal skin.^41^ Developing skin may therefore foster an environment for immune cell differentiation and maturation, much like lymphoid tissues.

### B‐cells

B‐cells have not been observed in fetal skin by flow cytometry^7^ or immunofluorescence^8^ using CD19 and CD20 as markers. However, using scRNAseq, B‐cells and their precursors have been detected in skin as young as 8 PCW.^41^ Detection of cells in various stages of B‐cell differentiation in first‐trimester skin indicates that B‐cell precursors are released from the liver into circulation where they can then seed tissue. It is yet to be determined whether the precursors differentiate within skin after seeding, or if specific stages are seeded, but the presence of B‐cell precursors without differentiated B‐cells in matched kidneys^41^ supports the former. Furthermore, it suggests the B‐cells were not merely present within the vasculature. Plasma cells, but not B‐cells, are present in steady‐state adult skin,^8^, ^59^, ^60^ so it is possible that B‐cells and their precursors may play a specific role during the development of fetal skin, or they may non‐specifically seed tissues, but this remains unknown.

## Mast cells, erythrocytes and megakaryocytes

Mast cells have been detected in fetal skin as early as 8 PCW,^41^ and continue to mature and expand throughout the second trimester.^8^, ^39^ This may be supported by the relatively high levels of stem cell factor, a molecule that induces mast cell chemotaxis and supports their differentiation,^61^ produced by embryonic skin compared with adult skin.^7^ Similar to mast cells in adult skin, they are commonly located near blood vessels and appendages,^39^ but at a significantly lower frequency.^8^, ^39^ Mast cells in first‐ and second‐trimester fetal skin contain immature granules,^39^, ^62^ which is reflected in the delayed detection of positive tryptase or toluidine blue staining in the second trimester,^8^, ^39^ despite tryptase transcripts being detected from 8 PCW^41^ and the presence of a distinct population of CD117^+^ mast cells being detected from 10 to 12 PCW.^39^ Mature chymase^+^ mast cells are not present until the end of the second trimester, and even then they are scarce in comparison to adult skin in which approximately 50% of mast cells are chymase^+^.^39^

As well as mast cells, there have also been megakaryocytes, erythroid cells and their common precursors have also been identified in first‐trimester skin.^41^ Furthermore, the erythroid cells detected were in various stages of development.^41^ This and the presence of their common precursor suggest that not only do mast cells differentiate in developing human skin, but physiological erythropoiesis may also be occurring.

## Role of immune cells during skin development

Establishment of what will become a diverse and extensive immune system in mature adult skin begins in the embryo, and continues to develop and expand *in utero*. Mature adult skin is constantly exposed to the external environment, which is reflected in the multitude of resident or circulating immune cells that provide continuous surveillance against potential threats.^60^ Conversely, pre‐natal skin develops in what is presumed to be a sterile environment.^63^, ^64^ As such, a surveillance role may be less essential, which is reflected by the reduced frequency of immune cells in developing skin compared with adult skin.^7^, ^8^, ^9^, ^10^, ^39^, ^53^ Despite containing relatively few immune cells, the immune component of developing skin is able to respond to stimuli. APCs in developing skin are able to efficiently take up antigens, upregulate co‐stimulatory molecules, stimulate T‐cell proliferation and migrate out of skin.^7^, ^38^ This suggests that an appropriate immune response could be mounted if challenged.

A key consideration that has not been fully explored is the role of immune cells in shaping the development of cutaneous microanatomical structures, including appendages such as hair follicles. The role of macrophages in tissue remodelling,^65^, ^66^ Tregs in hair follicle regeneration and maintenance,^67^ and interaction between immune cells and epidermal stem cells^68^, ^69^, ^70^ have been reported in postnatal murine life. The contribution of immune cells to skin generation during development will be an interesting avenue for future studies.

The presence of relatively fewer adaptive immune cells in developing versus mature adult skin in part reflects the timeline of haematopoiesis during development. Seeding of T‐cells and expansion of DC populations coincide with thymic and bone marrow development, respectively.^40^, ^49^, ^50^, ^51^ Prior to their contribution, expansion of the immune compartment in developing skin is compensated by high numbers of proliferating cells.^7^ The proportion of proliferating immune cells in embryonic skin is more than four times that of fetal skin, which in turn contains more than four times the number of proliferating immune cells than those found in mature adult skin.^7^ As such, the immune compartment in skin continues to expand both in number and diversity throughout development, although the longevity of these cells remains unclear. In mice, it has been shown that fetal LCs persist into adulthood,^12^ but whether the same occurs in humans and in the multitude of other immune cell types present in both fetal and adult skin is unknown. If immune cells seeded in fetal skin persist into adulthood, it is of interest to determine whether the function of any of these long‐lived populations evolved to reflect the needs of adult skin.

**Figure 2:**
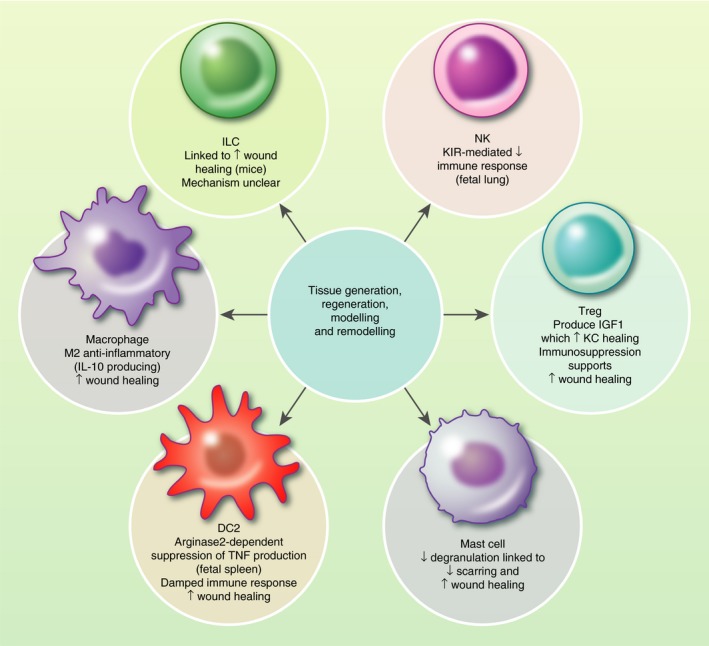
Summary of the role immune cells play during development of human skin. ↓, decreased; ↑, increased; DC, dendritic cell; IGF1, insulin growth factor 1; ILC, innate lymphoid cell; KC, keratinocyte; KIR, killer cell immunoglobulin‐like receptor; NK, natural killer cell.

The developing skin not only contains relatively fewer immune cells than mature adult skin,^7^, ^8^, ^9^, ^10^, ^39^, ^53^ but the cellular and chemical environment is skewed toward keeping the immune cells in an immunosuppressed or immunologically inert state as well (Fig. 2). Developing skin produces lower levels of pro‐inflammatory cytokines,^7^, ^71^, ^72^ and 18‐to 50‐fold higher levels of immunosuppressive cytokines than healthy adult skin.^7^ Other immune cells contribute to this, as seen in the skew toward anti‐inflammatory M2 macrophages,^7^, ^8^, ^44^ and potently immunosuppressive Tregs.^56^ Fetal lung NK cells are hypo‐responsive and express multiple inhibitory receptors,^57^ and fetal splenic DCs, which are transcriptionally similar to fetal skin DCs, are immunosuppressive in an arginase‐2 dependent manner,^38^ suggesting NK cells and DCs in developing skin may also contribute to the immunosuppressive environment. Contribution of multiple cell types, both immune and non‐immune, to maintaining an immunosuppressive environment indicates these interactions are crucial for normal development of skin.

Despite keeping immune cells immunosuppressed/immunologically inert, developing skin fosters immune cell differentiation. LCs differentiate and mature within the epidermis,^3^, ^7^, ^11^, ^25^ which is supported by TGF‐β1 production by the periderm and keratinocytes.^7^ The presence of megakaryocyte‐erythrocyte‐mast cell progenitors (MEMPs), lymphocyte precursors, B‐cell progenitors, ILC precursors and their progeny^41^ indicates differentiation of these lineages may be supported within developing dermis. This is also seen in the myeloid compartment, with the detection of myeloid precursors, monocytes and monocyte‐DC intermediates in first‐trimester skin.^41^ Fostering immune cell differentiation may be one method to aid expansion of the immune compartment of developing skin. Furthermore, tissue‐specific signatures have been reported for multiple immune cell types,^41^, ^42^, ^73^, ^74^ and differentiation within the tissue, as seen in skin, may contribute to the development of these signatures.

Skin goes through significant structural changes during development, which may contribute to the frequency and function of its immune compartment. Anatomically, there is no difference in the vasculature of second‐trimester fetal skin versus mature adult skin.^8^, ^75^ While this suggests that access to skin is not a limiting factor in seeding the immune network in second‐trimester fetal skin, it cannot be excluded that endothelial cells in fetal versus adult skin differ functionally, potentially influencing immune infiltration. Mature skin is rich with hair follicles and glands,^76^, ^77^ which only start to develop toward the end of the second trimester.^3^ The unique cells within these environments may provide crucial factors needed to maintain the immune cells that inhabit them, such as LCs in the epidermis^78^ and Tregs in hair follicles.^9^ Therefore, the establishment and development of some immune cells may be inhibited by the absence of their microanatomical niche.

The structure and properties of skin and the influence on immune development goes both ways. This is particularly evident when looking at wound healing. Developing human skin has the ability to heal without scarring, a feature that is restricted to the first and second trimesters.^79^, ^80^, ^81^ A key factor of scarless healing is reduced immune infiltrate,^6^, ^8^, ^81^, ^82^ therefore the immunosuppressive/immune inert environment and fewer immune cells in developing skin may contribute to this.^8^, ^79^, ^80^, ^81^ In mice, reduced scarring is observed in wounds containing fewer and immature mast cells, and is attributed to decreased degranulation.^83^ A similar mechanism may be operational in developing human skin, in which the mast cells contain immature granules.^39^, ^62^ Other immune cells linked to wound healing are T‐cells, ILCs and macrophages. In response to wounding, epidermal T‐cells have been shown to produce insulin‐like growth factor 1,^84^ which enhances would healing through regulation of keratinocytes,^85^ and ILC recruitment to cutaneous wounds in mice has been shown to promote wound healing, although the mechanism is unclear.^86^, ^87^ Macrophages in developing skin are skewed toward an M2 anti‐inflammatory phenotype, which is more supportive of wound healing,^46^ in contrast to M1 macrophages, which inhibit wound healing.^88^, ^89^ As such, these may also be contributing to scarless wound healing in developing skin. Appendage formation involves dynamic changes to the epidermis and dermis,^3^, ^4^, ^5^, ^90^ and perhaps immune cells involved in wound healing also aid the structural changes occurring during human skin development.

## Summary

Encased in a normally sterile environment, developing human skin begins to establish its immune system. In many of the immune cell populations identified, signs of differentiation within skin are also observed, indicating that the developing human skin is an environment that supports differentiation, similar to haematopoietic tissues. There is a diverse range of immune cells present in the developing human skin, many of which are immunologically inert or geared toward immunosuppression, wound healing, and potentially modelling the skin microarchitecture and appendage development. Better understanding of how the immune cells in developing skin function and interact, and how this contributes to tissue generation, superior wound healing and immunoregulation could lead to breakthroughs in inflammatory skin diseases, such as dermatitis, and regenerative medicine.

## Funding

M.H. and R.B. are funded by Wellcome (WT107931/Z/15/Z), The Lister Institute for Preventative Medicine and Newcastle‐Biomedical Research Centre.

## Disclosures

None to declare.
